# *Streptomyces*: The biofactory of secondary metabolites

**DOI:** 10.3389/fmicb.2022.968053

**Published:** 2022-09-29

**Authors:** Khorshed Alam, Arpita Mazumder, Suranjana Sikdar, Yi-Ming Zhao, Jinfang Hao, Chaoyi Song, Yanyan Wang, Rajib Sarkar, Saiful Islam, Youming Zhang, Aiying Li

**Affiliations:** ^1^Helmholtz International Lab for Anti-Infectives, Shandong University-Helmholtz Institute of Biotechnology, State Key Laboratory of Microbial Technology, Shandong University, Qingdao, China; ^2^Department of Microbiology, University of Chittagong, Chittagong, Bangladesh; ^3^Industrial Microbiology Research Division, BCSIR Chattogram Laboratories, Bangladesh Council of Scientific and Industrial Research (BCSIR), Chattogram, Bangladesh; ^4^Chinese Academy of Sciences (CAS) Key Laboratory of Quantitative Engineering Biology, Shenzhen Institute of Synthetic Biology, Shenzhen Institute of Advanced Technology, Chinese Academy of Sciences, Shenzhen, China

**Keywords:** *Streptomyces*, natural products, bioactive compounds, genome mining, metagenomics

## Abstract

Natural products derived from microorganisms serve as a vital resource of valuable pharmaceuticals and therapeutic agents. *Streptomyces* is the most ubiquitous bacterial genus in the environments with prolific capability to produce diverse and valuable natural products with significant biological activities in medicine, environments, food industries, and agronomy sectors. However, many natural products remain unexplored among *Streptomyces*. It is exigent to develop novel antibiotics, agrochemicals, anticancer medicines, etc., due to the fast growth in resistance to antibiotics, cancer chemotherapeutics, and pesticides. This review article focused the natural products secreted by *Streptomyces* and their function and importance in curing diseases and agriculture. Moreover, it discussed genomic-driven drug discovery strategies and also gave a future perspective for drug development from the *Streptomyces*.

## Introduction

### Diversity, morphology, taxonomy, and genetics of *Streptomyces*

The genus S*treptomyces* includes spore-forming, filamentous, and Gram-positive bacteria in Actinobacteria phylum (Khadayat et al., 2020; Lee et al., 2020), residing in a variety of environments, such as extreme environments and underexplored habitats, terrestrial and marine regions, symbionts, endophyte, mangroves etc. Over 850 species of *Streptomyces* have been studied (Kemung et al., 2018; Aryal et al., 2020). A gram of soil is estimated to contain 10^9^ colony-forming units (CFU) of bacteria, with 10^7^ CFU of Actinobacteria (Harir et al., 2018). S*treptomyces* species can form extensive branching substrate and aerial mycelia (Chater, 2016) and possess complex morphological characteristics with a complex multicellular development starting from the germination of hyphae from spores and ending with the formation of septa with a chain of uninucleated spores.

*Streptomyces* have an exceptionally high G + C% (>70%) and linear, moderately large genomes (8-10 Mb), regarded as special features among bacteria (Hopwood, 2019). *Streptomyces* have multiple biosynthetic gene clusters (BGCs) on each genome, which are the source of numerous bioactive compounds with medical or agricultural use (Ward and Allenby, 2018; Nicault et al., 2021).

### Necessities of new natural products

Antimicrobial resistance (AMR), one of the most severe public health challenges nowadays, is caused by resistant pathogenic bacteria, as well as fungi, viruses, and parasites (Amann et al., 2019). Every year, it causes deaths of 17 million people worldwide, along with a huge number of children and the elderly (Procópio et al., 2012). Multidrug resistant bacteria, some known as “superbugs,” make the conditions even worse. In the last few decades, the emergence of multidrug resistant bacteria increased devastatingly. The most common multidrug resistant bacteria are *Escherichia coli* resistant to cephalosporin and fluoroquinolones, *Klebsiella pneumoniae* to cephalosporin and carbapenems, *Staphylococcus aureus* to methicillin and vancomycin, *Neisseria gonorrhoeae* to cephalosporin, non-typhoidal *Salmonella* to fluoroquinolones and extended spectrum beta lactamase (ESBL), *Streptococcus pneumoniae* to penicillin, *Shigella* species to fluoroquinolones, and *Mycobacterium tuberculosis* to rifampicin, isoniazid, fluoroquinolone, and carbapenemase-producing enterobacteriaceae. The core reason for the recent increase in the antibiotic resistance phenomenon is indiscriminate and irrational use of antibiotics, poor hygiene and sanitation, frequent and self-medication, and poor control system and surveillance. The emergence and spreading of resistance mechanisms among bacteria are faster than the discovery and development of new antibiotics.

In addition, some non-negligible life-threatening diseases that enforce severe death worldwide are awaiting new drugs, including cancers as major causes of death in most of countries (Rotow and Bivona, 2017; Perin et al., 2022) and Alzheimer’s disease, which still has no effective drugs discovered and has caused dementia in 13.2 million patients in China by 2019 and will reach 150 million globally by 2050. In recent 30–40 years, some new infective diseases have fluently emerged to humans, including HIV, SARS, and Zika virus. In the last two and half years, SARS-CoV-2 has been causing serious problems for the human health as a pandemic worldwide. Specific and effective drugs for these emerging diseases have been yet underway (National Institutes of Health, 2007). The agrochemical field is also awaiting new generation of drugs, although limited types of agrochemicals, including avemectin or spinosad and glyphosate-derived compounds, are used widely.

In addition to the possibility of combining failing drugs with other compounds that appear to restore the desired effects (Brown, 2015), screening novel natural products, especially from *Streptomyces*, offers a viable alternative and an opportunity to improve the efficacy of treatments for these diseases, as an utmost significant solution to lessen this dreadful outcome.

## Natural products from *Streptomyces*

*Streptomyces* species have been considered as a repository of a diverse range of natural products (Azerang and Sardari, 2017; Genilloud, 2017; Lapaz et al., 2019; Xia et al., 2020) because of their powerful and complex secondary metabolism (Chang et al., 2021). *Streptomyces* produce around 100,000 antibiotic compounds, which account for 70–80% of all natural bioactive products with pharmacological or agrochemical applications (Bubici, 2018; Harir et al., 2018; Abdel-Razek et al., 2020).

*Streptomyces* produce a variety of natural products with high structural diversity, including macrolides, tetracyclines, aminoglycosides, glycopeptides, ansamycins, and terpenes. For example, *Streptomyces hygroscopicus* secretes around 180 metabolites with a wide range of bioactivities.

*Streptomyces-*derived bioactive natural products have the capability to function as antimicrobial, antiviral, cytotoxic, antitumor, antihypertensive, immunosuppressive, insecticide, antioxidative, plant growth-promoting, and herbicidal agents (Singh and Khajuria, 2018; Nguyen et al., 2020; Salwan and Sharma, 2020; Pham et al., 2021; Sharma et al., 2021). So, the discovered metabolites can be classified into four classes: (i) Compounds with regulatory activities include morphogenic agents, siderophores, and growth factors; (ii) anti-protozoans, antibacterials, antifungals, and antivirals as antagonistic agents; (iii) insecticides, pesticides, and herbicides as agrobiologicals; and (iv) neurological agents, immunomodulators, antitumor agents, and enzyme inhibitors as pharmacological drugs.

*Streptomyces* produce these compounds as secondary metabolites during the stationary phase, which are not necessarily required for their development or multiplication but can provide a competitive advantage to the organisms. Some of these metabolites aid reproductive bacterial cells by confiscating metals like iron (siderophores), shielding them from UV light (*via* pigmentation), limiting competition (antibiotics), and allowing them to communicate with other species, mediate important host–microbe and microbe–microbe interactions in mammalian or animal guts, and regulate biosynthetic pathways (Bolourian and Mojtahedi, 2018b; Wang et al., 2019). Furthermore, they play a vital role in soil biodegradation and in humus formation and produce numerous volatile substances, such as geosmin, which is responsible for the feature “wet earth odor.” Here, we discuss the essential products of *Streptomyces* rendering antibacterial, antifungal, antiviral, anti-immune, and anticancer properties and several important agrochemicals.

### *Streptomyces*-derived bioactive products with antagonistic or pharmacological properties

#### Antibacterial compounds with high diversity

**FIGURE 1:**
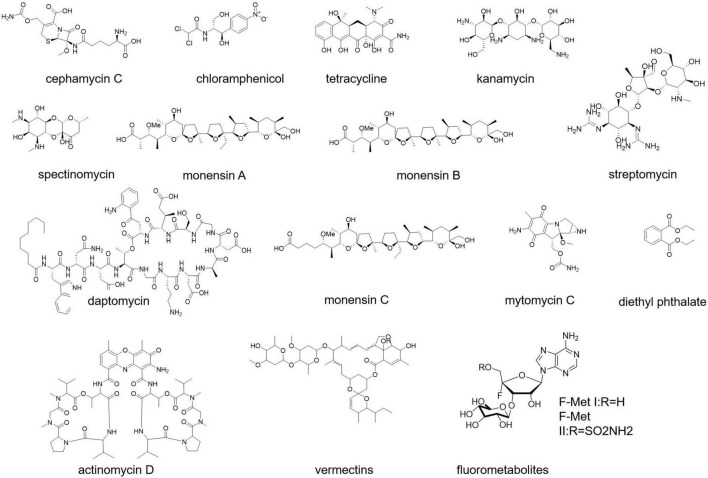
Antibiotics from *Streptomyces*.

Antibiotics are the most valuable products of *Streptomyces*. Actinomycetes produce two-thirds of all microbial antibiotics, with roughly 80% derived from the *Streptomyces* genus, which are well known for their therapeutic and agricultural uses. The most widely used antibiotics are derived from *Streptomyces*, from streptomycin first developed from soil isolates (Waksman, 1953) to well-known daptomycin (Figure 1) approved in 2003 by the FDA (Waters and Tadi, 2021). Terrestrial, marine, and estuarine environments are niches for the storage of antimicrobial drugs for *Streptomyces*. Almost half of the *Streptomyces* species isolated have been identified as antibiotic producers.

Some popular antibiotics, for example, cephamycin, chloramphenicol, tetracycline, kanamycin, spectinomycin, monensin, and mitomycin C (Figure 1) have been originated from the species of *S. clavuligerus, S. venezuelae, S. aureofaciens, S. kanamyceticus, S. spectablis, S. cinnamonensis*, and *S. lavendulae*, respectively (Akintunde, 2014). *S. griseus, S. avermitilis*, and *S. cattleya* produce streptomycin, polypeptide avermectin, and fluorometabolites (Figure 1), respectively (Ikeda et al., 1999; Nicault et al., 2021). Other than that, there are also some other species of *Streptomyces* that can produce a good number of antibiotics. A new strain *S. parvulus* strain MARS-17, found in the soil of Rajshahi, Bangladesh, secretes actinomycin D (Figure 1; Rahman et al., 2011). A study showed that n-butanol extracts of *S. flavogriseus* strain ACTK2 exhibit a broad spectrum of antimicrobial activity (Dezfully and Ramanayaka, 2015). *S. cheonanensis* VUK-A strain secretes two bioactive compounds 2-methyl butyl propyl phthalate and diethyl phthalate (Figure 1) possessing a broad range of antimicrobial activity (Dezfully and Ramanayaka, 2015).

Nowadays, several *Streptomyces* species are accountable for both medicinal and commercial antibiotics, and they perform magnificently in these contexts. Many antibiotics have been accumulated in one species of *Streptomyces* (e.g., *S. griseus* and *S. hygroscopicus*), as well as the same antibiotics are produced from different *Streptomyces* species (e.g., streptothricin and actinomycin).

Antibiotics are usually grouped together based on their action mechanism, chemical structure, or spectrum of activity. Antibiotics derived from *Streptomyces* are categorized according to their principal structural classes. Major types of antibiotics produced by *Streptomyces* are aminoglycosides (such as gentamicin, streptomycin, tobramycin, neomycin, and kanamycin), anthracyclines (doxorubicin), glycopeptides, β-lactams (monobactams, cephalosporin, and carbapenems), macrolides (clarithromycin, erythromycin, and azithromycin), ansamycins (rifamycin), nucleosides, peptides, polyenes, polyesters, and tetracyclines.

##### Antibacterial compounds of *Streptomyces* in extreme environments

Most of bioactive compounds were isolated from conventional terrestrial environments, mentioned as before. *Streptomyces* isolated from extreme environments also have the capability to generate some antibacterial products, including alkaloids, angucyclines, macrolides, and peptides (Sivalingam et al., 2019). Some strains of natural bioactive antibacterial metabolite secretors are *Streptomyces* sp. RAUACT-1, *S. arenicola, S. griseus, S. nodosus, S. lincolnensis, S. pacifica*, and *S. pristinaespiralis* secreting 1,4-dihydroxy-2-(3-hydroxybutyl)-9,10-anthraquinone, anthracene 9,10 anthracene, arenimycin, frigocyclinone, lajollamycin, lincomycin, mitomycin C, pacificanones A and B, pristinamycin, and rapamycin, respectively. *Streptomyces* secrete bisanthraquinone, carbomycin, glaciapyrroles, and tirandamycins that also display antibacterial activity (Harir et al., 2018). Recently, thermophilic *Streptomyces werraensis* MI S.24 3, isolated from a very harsh environment in Egypt, has both antibacterial and cytotoxic properties (Mohamed et al., 2021).

The production of one-of-a-kind polyketides and phenolic compounds by *Streptomyces lanatus* AR2 with observable bioactivities made it possible for the organism to adapt to harsh environment like salty wetland, yielding useful natural products with biotechnological, pharmaceutical, and medical applications (Riahi et al., 2019).

Marine *Streptomyces* are considered the largest and an outstanding repository of myriad natural products and antibiotics. Marine *Streptomyces* are found in different aquaculture, such as in seaweeds, mollusks, fishes, sponges, mangroves, seawater, and sediments (Dharmaraj, 2010).

**FIGURE 2:**
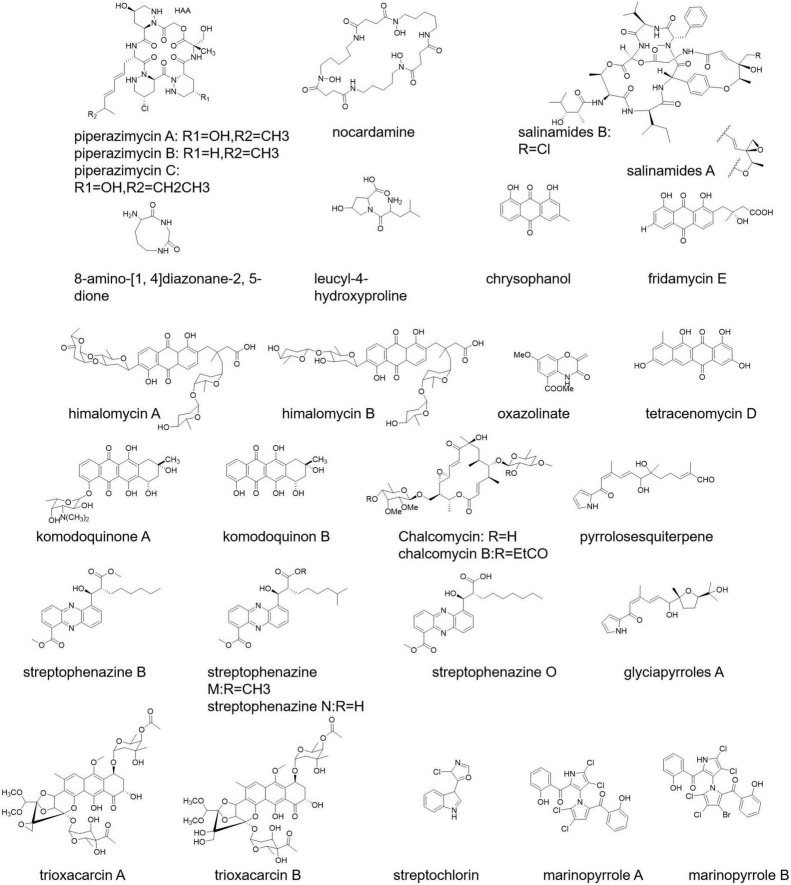
Bioactive compounds from marine *Streptomyces*.

Piperazimycin A-C, allophycocyanin, nocardamine, salinamides A and B, 8-amino-[1,4] diazonane-2, 5-dione, and leucyl-4-hydroxyproline are some of the marine-originated polypeptide antibiotics (Figure 2) isolated from *Streptomyces* strain KMM7210, *Streptomyces* strain M097, *Streptomyces* sp. (from an unidentified marine sponge), *Streptomyces* sp. CNB-091, and *S. acrimycini*, respectively (Hernández et al., 2004; Hou et al., 2006; Sobolevskaya et al., 2007; Dharmaraj, 2010).

*Streptomyces* sp. 6921 from marine sediments of Mauritius secretes C-glycosides himalomycins, anthraquinones, fridamycin E, and chromophore (Figure 2) possessing strong antibacterial activity (Maskey et al., 2003). *S. corchorusii* AUBN secretes tetracenomycin D (Figure 2) having cytotoxic activity (Adinarayana et al., 2006). Komodoquinone A, B (Figure 2), anthracycline antibiotics, are produced from the fermentation broth of *Streptomyces* sp. KS3 showing neurogenic activity (Itoh T. et al., 2003).

Chalcomycin and chalcomycin B (Figure 2) are macrolides isolated from *Streptomyces* sp. M491 and *Streptomyces* isolate B7064 (Wu et al., 2006). *Streptomyces* sp. NPS008187 produced pyrrolosesquiterpenes and glyciapyrroles A (Figure 2) possessing strong antibacterial activity (Macherla et al., 2005). The product streptophenazines (Figure 2) from marine *Streptomyces* sp. 182SMLY works against a methicillin-resistant *S. aureus* (Liang et al., 2017). Another example is marinopyrroles (Figure 2; Al-Ansari et al., 2019).

A new tetracycline analog SBR-22, a polyketide, isolated from *S. psammoticus* exhibits antibacterial activity against methicillin-resistant *S. aureus* (MRSA) (Sujatha et al., 2005). Trioxacarcin A, B, and C (Figure 2) show anti-plasmodial activity, and these products have been secreted by *S. ochraceus* and *S. bottropensis* (Dharmaraj, 2010). Streptochlorin (Figure 2) from the *Streptomyces* strain 04DH110 possesses antiproliferative activity (Shin et al., 2007).

A strain of *Streptomyces olivaceus* isolated from a mangrove in Macau, China, was subjected to whole-genome sequencing and MS/MS analysis to determine the strain potential to produce secondary metabolites. According to the findings, the genome of *S. olivaceus* had a total of 105 gene clusters, and genome mining was able to predict the presence of 53 known secondary metabolites. There were 28 secondary metabolites that were classified as antibiotics and derived from *S. olivaceus*; however, none of them had been discovered before (Hu et al., 2021).

##### Antibacterial compounds against biofilm formation in “superbugs”

Biofilm formation is considered to be one of critical factors to determine the toxicity of some pathogenic bacteria, including well-known MRSA. *Streptomyces*-derived numerous antibiotics or their derivates were used for antibiofilm activity in MRSA. A total of three antibiotics, namely, rifampin, daptomycin, and tigecycline were exploited for the treatment of device-related *Staphylococcal* biofilm infections such as antimicrobial lock therapy (Hogan et al., 2016). Daptomycin, tigecycline, and rifampin showed better antibiofilm activity against mature biofilms, whereas vancomycin failed to eradicate older biofilms in model experiments (Hogan et al., 2016). Minocycline is also found to be effective compared with vancomycin in inhibiting biofilm formation and eliminating mature biofilms in ica-positive and ica locus-negative MRSA (Chopra et al., 2015).

**FIGURE 3:**
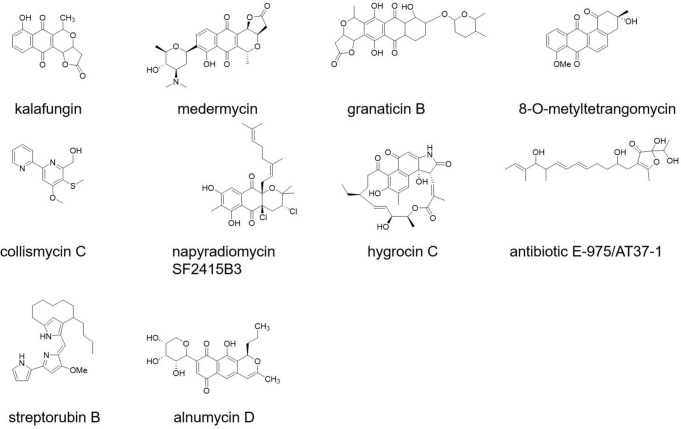
Compounds with anti-methicillin-resistant *Staphylococcus* aureus (MRSA) biofilm activity.

Some newly discovered bioactive compounds from *Streptomyces* species showed promising antibiofilm activities against *S. aureus* and MRSA (at the micromolar level), including antibiotic 5812-A/C (Vasilchenko et al., 2020), streptorubin B (Suzuki et al., 2015), alnumycin D, granaticin B, kalafungin, medermycin (Oja et al., 2015), collismycin C, napyradiomycin SF2415B3 (Bauermeister et al., 2019), hygrocin C (Wang et al., 2018), 8-O-metyltetrangomycin (Mary et al., 2021), panglimycin D (Kim et al., 2020), and AT37-1 (Figure 3; Driche et al., 2017).

#### Antifungal products from *Streptomyces*

The resurgence of fungal infection is a public health crisis. Secondary metabolites produced by Actinobacteria, particularly members of the *Streptomyces* genus, are known to have a wide range of biological activities, including antifungal compounds (Siupka et al., 2021).

**FIGURE 4:**
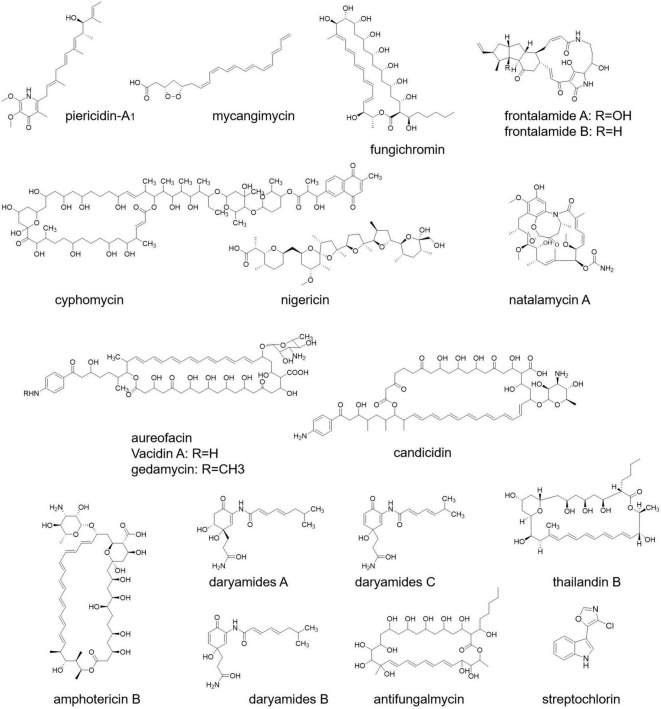
Antifungal compounds from *Streptomyces*.

According to a study, *Streptomyces* sp. ICBG292, isolated from the exoskeleton of *Cyphomyrmex* workers, produces piericidin-A_1_ and nigericin (Figure 4), which inhibit the growth of fungal pathogen *Escovopsis* and also prevent the infection of *Leishmania donovani*, a human pathogen (Ortega et al., 2019). In another research, it has been found that pathogenic fungus *Rhizoctonia solani* AG-4 deteriorating the condition of the leaf of cabbage can be treated by *S. padanus* strain PMS-702. The responsible compound for this fungal inhibition is the polyene macrolide fungichromin (Figure 4), which possesses strong antifungal activity (Shih et al., 2003).

A new molecule named cyphomycin (Figure 4) possesses antifungal activities against multidrug-resistant fungal pathogens (Chevrette et al., 2019). *Streptomyces* symbiotically with southern pine beetle (*Dendroctonus frontalis*) secretes the secondary metabolites mycangimycin, frontalamide A, and frontalamide B (Figure 4). Frontalamide possesses antifungal activity and mycangimycin against the growth of the antagonistic fungus *Ophiostoma minus* (Chevrette et al., 2019). Streptochlorin and natalamycin derived from *Streptomyces* also exert antifungal activity (Chevrette et al., 2019). Other than that, *S. nodosus, S. aureofaciens, S. griseus, S. noursei, S. aureus*, and *S. diastachromogenes* exert antifungal compounds named amphotericin, aureofacin, candicidin (Figure 4), nystatin, oligomycin, and actinomycin D, respectively (Akintunde, 2014).

Daryamide (Figure 4) is another antifungal compound of *Streptomyces* (Fu et al., 2017). A strain of *Streptomyces* identified from rhizospheric soils secretes four antifungal compounds, namely, actinomycin, fungichromin, thailandin B, and antifungalmycin (Figure 4; Peng et al., 2020). In a study, it has been shown that *S. halstedii* K122 exhibits antifungal compounds bafilomycin B1 and C1, which inhibit the growth of fungi *Penicillium roqueforti, Aspergillus fumigatus, Paecilomyces variotii*, and *Mucor hiemalis* (Frändberg et al., 2000). In another study, antifungal protein 1 (Afp1) from *S. tendae* is used to limit fungal growth (Freitas et al., 2005).

#### Antiviral compounds from *Streptomyces*

**FIGURE 5:**
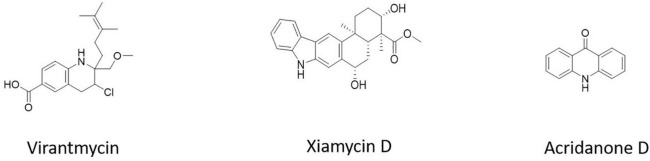
Antiviral compounds from *Streptomyces*.

Virantmycin B (1) (Figure 5), isolated from *Streptomyces* sp. AM-2504, showed activity against the dengue virus (Hirota-Takahata et al., 2017). The bioactive chemical xiamycin D (Figure 5), isolated from *Streptomyces* sp. (#HK18) culture, had the strongest inhibitory effect on PEDV proliferation (Hayakawa et al., 2013). 9(10H)-acridanone (Figure 5), a secondary metabolite isolated from *S. fradiae* strain VITMK2, could inhibit the white spot syndrome virus (WSSV) (Hayakawa et al., 2013).

Dichloromethane extracts (DCME) of *Streptomyces* sp. demonstrated broad and potent antiviral activity against a variety of influenza viruses, including H1N1 and H3N2 subtypes, and influenza B virus. DCME may interact with the HA2 subunit of influenza A virus (IAV) hemagglutinin (HA) *via* stopping the fusion process between the viral and host cell membranes, preventing the virus from entering host cells, according to a thorough mode-of-action investigation (Zhao et al., 2019). *Streptomyces* sp. SMU03 produced an antiviral butanolide [(4S)-4-hydroxy-10-methyl-11-oxo-dodec-2-en-1,4-olide] that has broad and robust activity against influenza viruses, including H1N1 and H3N2 subtypes, and influenza B virus (Li et al., 2018).

#### Immunostimulant, anti-immunosuppressive, and vasoactive substances from *Streptomyces*

There are thousands of natural substances that influence immune cell activity or antibody secretion to manage infection and maintain immunological homeostasis and thus regulate the immune system. Immunostimulants are a diverse collection of chemicals that act on the immune system in a non-specific way by activating it, either by upregulating it or by favoring the activity of one of its components (Ortuño-Sahagún et al., 2017). Secondary metabolites from *Streptomyces* bacteria have a wide range of immunomodulatory activity made a significant contribution to immunomodulatory therapies (Mahmoudi et al., 2016).

**FIGURE 6:**
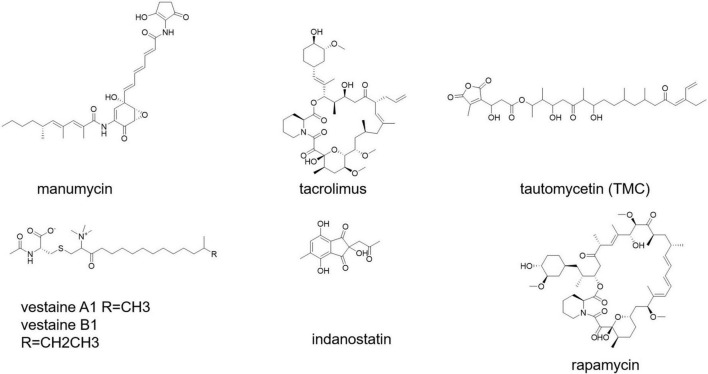
Immunostimulant, anti-immune suppressive and vasoactive compounds.

Secondary metabolites from *S. calvus* have been shown to be effective immunomodulators in human peripheral blood mononuclear cells (PBMCs) (Mahmoudi et al., 2016). Short polyketides generated by several streptomycetes and related bacteria that have strong anti-inflammatory and pro-apoptotic properties are known as manumycin-type compounds (Figure 6; Hrdý et al., 2020).

Immunosuppressants are used to prevent transplanted tissues and organs from being rejected, as well as to treat autoimmune illnesses (Fireman et al., 2004). Antiproliferative/immunosuppressive chemicals produced by *Streptomyces* bacteria (e.g., rapamycin and tacrolimus) (Figure 6) play an important role in the resistance of certain mucosal cancers (e.g., colon cancer) and the hygiene hypothesis (Bolourian and Mojtahedi, 2018a). Tacrolimus was discovered as a potent immunosuppressant in the strain *Streptomyces tsukubaensis* 9993T, which was first used in Japan in 1993 to reduce graft rejection after liver transplantation (Barreiro et al., 2012; Muramatsu and Nagai, 2013). Various taxonomically different *Streptomyces* strains, notably *S. tacrolimus* ATCC 55098T, have been identified as tacrolimus producers (Kim and Park, 2008; Martínez-Castro et al., 2011).

*Streptomyces* sp. CK4412 synthesized an activated T-cell-specific immunosuppressive chemical named tautomycetin (TMC) (Figure 6; Choi et al., 2007). TMC has a mechanistically distinct immunosuppressive action that was 100 times greater than that of cyclosporine A (Choi et al., 2017). TMC significantly suppressed breast cancer cell propagation, migration, and invasion, suggesting that it could be used as a possible breast cancer therapeutic alternative (Niu et al., 2013). Rapamycin and tacrolimus are antiproliferative/immunosuppressive drugs found in *Streptomyces*. *S. hygroscopicus* and *S. tubercidicus* probiotics are effective against inflammatory diseases (Bolourian and Mojtahedi, 2018a).

A vasoactive substance is an endogenous agent or pharmaceutical medicine that, by its vasoactivity, or vascular activity, increases or decreases blood pressure and/or heart rate (effect on blood vessels). *Streptomyces* sp. SANK 63697 produced two vasoactive chemicals, namely, vestaine A1 and vestaine B1 (Figure 6; Hirota-Takahata et al., 2017). *Streptomyces* sp. RAI20 yielded indanostatin (Figure 6), a neuroprotective compound against glutamate toxicity (Hayakawa et al., 2013).

#### Anticancer compounds from *Streptomyces*

**FIGURE 7:**
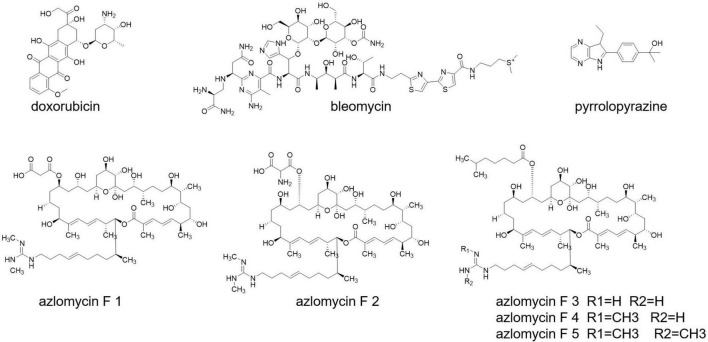
Anti-cancer drugs from *Streptomyces*.

Cancer is still a dreadful disease and causes millions of death every year. *Streptomyces* also produce some well-known anticancer drugs, such as doxorubicin and bleomycin (Grimm et al., 1994; Du et al., 2000; Figure 7). A research carried out with Malaysian mangrove forest revealed the presence of Malaysia phenolic and pyrrolopyrazine (Figure 7) compounds in *Streptomyces* having anticancer activities (Azman et al., 2017). *Streptomyces* sp. 211726, isolated from soil, also exerts anticancer compounds, with seven azalomycin F analogs (Figure 7) and macrocyclic lactones (Tan et al., 2015).

Anticancer activity has been notified from the product selina-4(14),7(11)-diene-8,9-dio from *Streptomyces* spp. QD518 (Wu et al., 2006). Amorphane and T-muurolol (Figure 7) are terpene antibiotics secreted by *Streptomyces* sp. M491 (Wu et al., 2007; Ding et al., 2009). T-Muurolol shows moderate cytotoxic activity (Ding et al., 2009). Daryamides A-C (Figure 7) from *Streptomyces* sp. CNQ-085 has anticancer and anticytotoxic activities (Asolkar et al., 2006). *Streptomyces* spp. BL-49-58-005 has been isolated from a marine invertebrate, and it secrets indoles A, B having cytotoxic activity.

### *Streptomyces*-derived bioactive compounds with agrobiologic properties

*Streptomyces* species regulate soil fertility by interacting with a variety of factors and act as nutrient enhancers, as an abundant source of bioactive secondary metabolites used in agriculture (Vurukonda et al., 2018), which are all impacted by chemical pesticides or insecticides with complex structures.

#### Insecticide, pesticide, and herbicide substances

The *S. hydrogenans* DH16 extract ethyl acetate had larvicidal and growth-suppressive properties (Kaur et al., 2014). *Streptomyces* produces avermectins, which have activity against mites, pear psylla, and diamond back moth (Lehman et al., 2004). *S. rimosus* could be used to reduce pesticide pollution in environments (Khajezadeh et al., 2020). A variety of cellulolytic and hydrolytic enzymes produced by *Streptomyces* may enter into the plant materials by rupturing the epidermal cell walls and middle lamellae between plant cells (Viaene et al., 2016). Spinosad from actinomyces *Saccharopolyspora spinosa* was registered by the EPA as a new chemical class of insecticides with a double glycosylated polyketide structure, and it was now accumulated heterologously at the highest yield (70 mg/L) in *Streptomyces albus*, while in *Streptomyces* hosts, pathway refactoring led to the production of some novel spinosad derivatives (Song et al., 2020; An et al., 2021).

#### Antifungal substances

**FIGURE 8:**
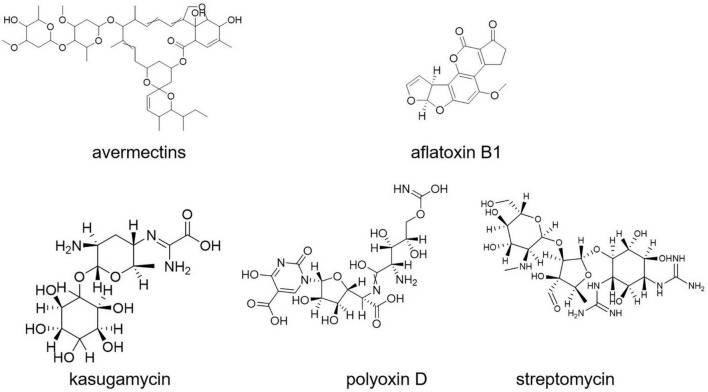
Insecticides, pesticides and herbicides compounds.

Aflatoxin B1 contamination is controlled with *S. roseolus* (Caceres et al., 2018). Similarly, *S. yanglinensis* could inhibit the formation of *Aspergillus flavus* mycelium in protecting food and feed from aflatoxin contamination (Shakeel et al., 2018; Lyu et al., 2020). *S. griseoviridis* K61 (Mycostop) was used for the biocontrol of black scorch on date palms caused by *Ceratocystis radicicola* and soilborne diseases, namely, *Fusarium oxysporum* sp. *lycopersici* (FOL) and verticillium of tomato (Suleman et al., 2002; Minuto et al., 2006). A total of four different strains of *Streptomyces* (viz., *S. violaceusniger* YCED9, *S. saraceticus* KH400, *S. lydicus* WYEC108, and *S. griseoviridis* K61) were used for biocontrol of fungal and bacterial soil diseases in nine (ix) different formulations. Kasugamycin, streptomycin, and polyoxin D (Figure 8) are three purified metabolites produced by *Streptomyces* sp. that are used as foliar fungicides and bactericides (Rey and Dumas, 2017). The synthesis of a thiopeptide that stops *Fusarium* cell wall biosynthesis could be linked to the disease-suppressive actions of *Streptomyces* strains, according to genome mining and mutant analysis (Cha et al., 2016; Rey and Dumas, 2017).

*Streptomyces* S4-7 was found to be resistant to *Fusarium* wilt disease (Kim et al., 2017). Culture extracts of *Streptomyces globisporus* JK-1 could inhibit *Magnaporthe oryzae* more effectively than tricyclazole, a commonly used chemical fungicide for the control of the rice blast fungus (Newitt et al., 2019). Kasugamycin (an antibiotic derived from *S. kasugaensis*, with brand name Kasumin in Japan) is used to prevent rice blast disease. Rice plants with transgenic expression of the *S. griseus* chitinase-encoding gene *chi*C showed improved resistance to the rice blast fungus *Magnaporthe grisea* (Itoh Y. et al., 2003).

### Natural products from endophytic *Streptomyces* species

*Streptomyces* species are unique evolutionally because they lie between fungi and bacteria. Endophytic *Streptomyces* species appear asymptomatically in animal or human hosts by participating in the physiological process in the animal body or guts. Most of endophytic *Streptomyces* from plants are involved in the rhizophagy cycle and improve plant growth, reduce oxidative stress, and suppress the growth of competitive plants (Hallmann et al., 1997; Bacon and White, 2000). Here, we mentioned some different endophytic *Streptomyces* species that can produce bioactive natural products.

**FIGURE 9:**
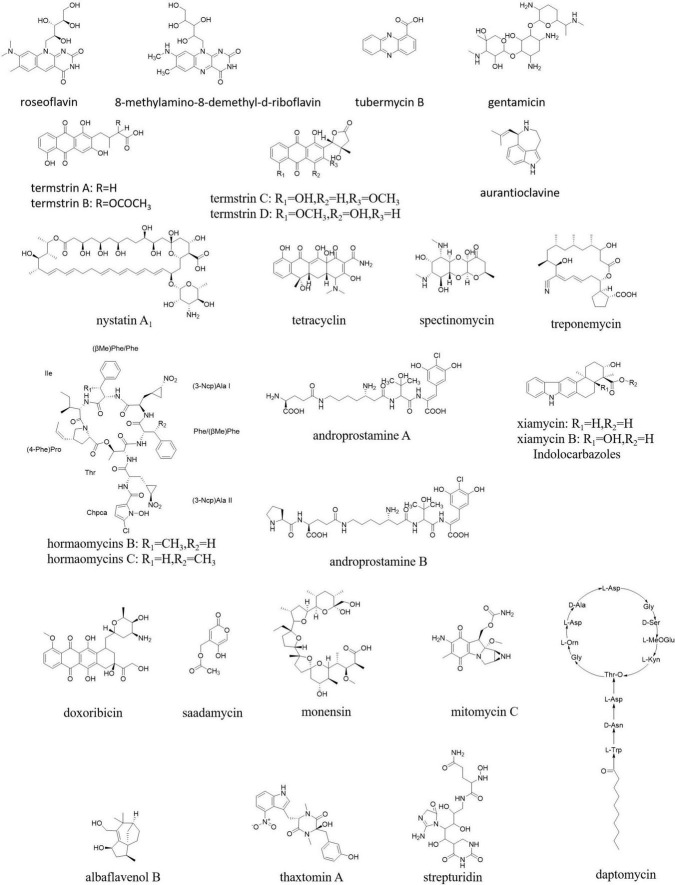
Natural products from endophytic *Streptomyces*.

A diverse group of pharmaceutical compounds (Figure 9) have been isolated from different endophytic *Streptomyces* species and have a wide range of activities, such as antibacterial, antifungal, antimalarial, anticancer, and larvicidal, including nystatin, munumbicin, hormaomycin, tetracycline, coronamycin, spectinomycin, treponemycin, androprostamines, indolocarbazoles, doxorubicin, anthracycline, daptomycin, monensin, mitomycin C, saadamycin, strepturidin, thaxtomin A, xiamycin, and albaflavenol B (Ayswaria et al., 2020).

Although *Streptomyces* in mammal guts are less prevalent than those in soils, among a limited number of *Streptomyces* identified as “old friends” in human guts, a species showed activity against colon cancer. So, it was deduced that such *Streptomyces* able to produce antiproliferatives/immunosuppressants could reduce colon cancer directly while residing in human guts as possible “old friends” (Bolourian and Mojtahedi, 2018b). A high number of natural products were identified from *Streptomyces* in the animal guts, especially in the termite guts. For example, roseoflavin and 8-methylamino-8-demethyl-d-riboflavin from *S. davaonensis* living in the body surface of the queen of *Odontotermes formosanus* strongly inhibited *Bacillus subtilis* and *S. aureus* (Zhou et al., 2021). Termstrins A, B, C, and D (anthraquinone derivatives) were produced by *Streptomyces* sp. BYF63 from termite guts; among them, termstrins A and C showed potent antibacterial activities against *S. aureus* (Zhang et al., 2020). A polyene-producing *Streptomyces* spp. from the fungus-growing termite *Macrotermes barneyi* showed strong bioactivity against the antagonistic fungus *Xylaria* (Li J. et al., 2021).

Overall, 12 isolates from 65 endophytic *Streptomyces* sp. were found to have antibacterial activity against penicillin-resistant *S. aureus* (Passari et al., 2017). An endophytic actinomycete isolated from an ethnomedicinal plant *Streptomyces kebangsaanensis* sp. nov. produced tubermycin B (phenazine-1-carboxylic acid) with antibacterial activity (Tan et al., 2013). *Streptomyces* sp. SUK was proved to produce non-toxic antibiotics, such as aurantioclavine and gentamicin. Endophytic *Streptomyces* spp. from maize had antibacterial and antifungal activities against many pathogens (de Araújo et al., 2000).

Endophytic *Streptomyces* spp. SUK 12 and SUK 48 extracts were found to be shown anti-plasmodial activity (Ahmad et al., 2021). A *Streptomyces* strain producing munumbicins A, B, C, and D was reported to inhibit malarial infection (Castillo et al., 2002). Shimizu et al. (2004) revealed antitumor agent actinomycin X2-producing endophytic *S. galbus* R-5 (Shimizu et al., 2004). Tanvir et al. (2014) reported the larvicidal effect of endophytic *Streptomyces* spp.

Many endophytic *Streptomyces* species could produce amylases and cellulases, especially extracellular amylases being widely used in the textile, distilling, food, and brewing industries. For example, endophytic alkali-thermotolerant *S. gulbargensis* DAS 131 was reported to produce amylases (Syed et al., 2009).

Some endophytic *Streptomyces* species were reported to accumulate phytohormones, antibiotics, and siderophores used to improve the fitness of the plants. Overall, three *Streptomyces* species, namely, *Streptomyces fulvoviolaceus, Streptomyces caelestis*, and *Streptomyces coelicolor*, showed strong activity against one or more pathogenic fungi, including *Phytophthora erythroseptica, Pythium ultimum, Sclerotinia sclerotiorum, Mycosphaerella fijiensis*, and *Rhizoctonia solani* (Zin et al., 2007). *Streptomyces* SUK 25 produced pharmaceutically active compounds (PhACs) in submerged fermentation using response surface methodology (RSM) as a tool for optimization (Al-Shaibani et al., 2021).

Some endophytic Streptomyces are able to produce biosurfactants. *Streptomyces* sp. DPUA1566 could produce biosurfactant production using corn steep liquor substrates (Santos et al., 2019). Marine endophytic *Streptomyces* spp. NRC50 and NRC51 were able to accumulate prodigiosin-like compounds (red pigments) using agrowaste materials as substrates (El-Bondkly et al., 2012). Some volatile organic products with antifungal activity from *Streptomyces setonii* WY228 could control black spot disease of sweet potato (Gong et al., 2022).

## Action modes of bioactive compounds from *Streptomyces* species

Different bioactive natural products from *Streptomyces* exert their activities *via* different mechanisms.

### Action modes of antibacterial compounds

**FIGURE 10:**
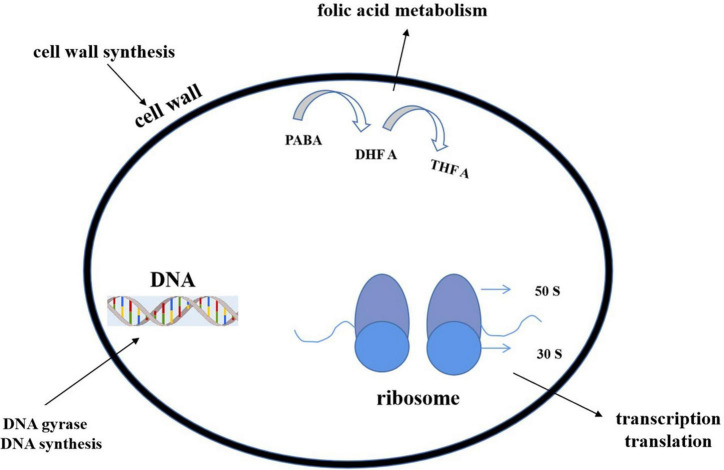
Action modes of antibacterial compounds. By targeting the bacterial cell wall/cell membrane, antibiotics inhibit bacterial growth. The synthesis of nucleic acids and proteins are two further targets. The latter is a function of ribosomes, which are nucleoprotein complexes made up of a small and large subunit (30 S and 50 S in bacteria, as shown in the figure). Antibiotics can also operate as antimetabolites by blocking folate metabolism (and thus DNA synthesis) through a pathway that includes para aminobenzoic acid (PABA) and two folic acid precursors, dihydrofolic acid (DHF) and tetrahydrofolic acid (THFA) (THF). Antibiotics can stop DNA gyrase from changing the shape of DNA, which is important for replication and transcription.

Most of the antibiotics act upon some specific parts of bacteria and stop the cellular processes or cellular synthesis. The specific targets include inhibition of (1) deoxyribonucleic acid (DNA) or ribonucleic acid (RNA) synthesis (DNA topoisomerase or RNA polymerase), (2) protein synthesis (ribosome), or (3) cell wall (peptidoglycan) synthesis (Figure 10).

#### Cell wall biosynthesis inhibition

Generally, the β-lactam group include penicillin, cephalosporin, and vancomycin. The first two antibiotics inhibit the cell wall precursor–peptidoglycan crosslink. A serine hydroxyl group attacks β-lactam rings and produces an acyl intermediate, which hydrolyzes very slowly, terminates cell wall synthesis, and lessens the number of reproducing cells (Walsh, 2003). Vancomycin binds with the network of strong hydrogen bonding and stops the cell wall generation process (Rossiter et al., 2017). Lipid II is an essential intermediate in peptidoglycan biosynthesis in bacteria. Some peptides (like some lanthiopeptides) could bind to lipid II to impair cell wall synthesis. Meanwhile, such binding also caused the formation of a polymerized complex, leading to perforation on cell membranes and electrolyte outflow (Li C. et al., 2021).

#### Inhibition of protein synthesis

Erythromycin, oxazolidinones including linezolid, and tetracycline, come under this category. Erythromycin and linezolid attack the larger 50S subunit of the ribosome (Levy and Marshall, 2004; Ruuskanen and Järvinen, 2014). Tetracycline binds the smaller 30S subunit and blocks the translation process (Rossiter et al., 2017).

#### Inhibition of DNA or RNA synthesis (DNA topoisomerase or RNA polymerase)

DNA and RNA replication have been blocked by rifampicin, quinolones, ciprofloxacin, etc. (Kohanski et al., 2010). They interfere with the DNA gyrase, unwind the both strands of DNA, bind with the cleavage site, and stop the process of further of DNA synthesis (Rossiter et al., 2017).

### Action modes of antifungal compounds

Fungal cell membranes have a unique sterol, ergosterol, differing from mammalian cell membranes (cholesterol). The chitin structured fungal cell wall is absent in human cell. Thus, for some selective antifungal agents, fungal cell walls may be considered to be prime targets (Sudhir et al., 2010).

Piericidin A1 showed antitumor/anti-insect activity by competitive binding to the mitochondrial complex I (Li et al., 2019). It was also found to have the most potent inhibitory activity against *Xanthomonas oryzae pv. oryzicola* and *Penicillium decumbens* (Shang et al., 2018; Li et al., 2019). Piericidin A and glucopiericidin A are also potential quorum sensing inhibitors (QSIs) that suppress the expression of Eca virulence genes of plant pathogen *Erwinia carotovora* subsp. atroseptica (Eca), implying that they could be used as soft rot disease control agents on potato tubers (Kang et al., 2016).

Nigericin can promote an exchange of K+ for H+, which changes the ion gradient across membranes involved in energetic metabolism and thus inhibits the growth of the *Leishmania* parasite (Ortega et al., 2019).

Fungichromin may inhibit the biofilm formation of *C. albicans* by downregulating the expression of the genes (ALS1, ALS3, HWP1, HYR1, EFG1, CPH1, and BCR1) (An et al., 2016). Streptochlorin mechanism of action for antifungal activity is unknown; however, a few studies have shown that it can inhibit the activity of monoamine oxidase (Jia et al., 2018).

Another antifungal agent, AFP1, as a novel member of the bg-crystallin superfamily, could change fungal morphogenesis. It was observed to bind to crab shell chitin, chitosan (deacetylated chitin), and the cell walls of the fungus *P. variotii* (Campos-Olivas et al., 2001).

### Action modes of antiviral compounds

Viruses are obligate intracellular parasites and have no cell walls or cell membranes. They do not have metabolic machinery of their own and have to use hosts’ enzymes. Targeting the viruses themselves and the host cell components are the two main techniques for developing antiviral drugs.

Antiviral compounds by inhibiting virus attachment, entrance, uncoating, genome replication, and inhibiting some viral enzymes (DNA polymerase, protease, nucleotide reverse transcriptase, and integrase) are some antiviral medications that specifically target viruses. Protease inhibitors including ritonavir, atazanavir, and darunavir; viral DNA polymerase inhibitors including acyclovir, tenofovir, valganciclovir, and valacyclovir; and integrase inhibitors (such as raltegravir) are among the top 200 drugs by sales in the 2010s. But, many viral infections still lack effective antiviral treatment.

Xiamycin D showed inhibition against PEDV RNA expression, nucleocapsid protein synthesis, and also lowering RNA levels. Its activity is associated with the GP2 spike protein, a key regulator in the viral entry step (Kim et al., 2016).

Some known antiviral drugs were found to enforce the cell resistance to a virus (interferons), block viral attachment, diffusion into the host cells (amantadine), or viral deproteinization process in the cell (amantadine). They also lower cell metabolisms to inhibit viral nucleic acid synthesis. Other antiviral agents, including butanolide, could prevent viral entrance into host cells by blocking the fusogenic process of viral attachment proteins, such as hemagglutinin (HA) of influenza A virus (IAV) (Li et al., 2018).

### Action modes of insecticide, pesticide, and herbicide substances

Chemicals known as “insecticides” are put to use to get rid of insects either by killing or by preventing them from carrying out harmful or unwanted behaviors. Based on the difference in structure and mode of action, insecticides are classified into organophosphates, pyrethroids, triazines, carbamates, and organochlorines. Many insecticides act upon the insect’s nervous system (e.g., cholinesterase inhibition), while others act as growth regulators or endotoxins.

Organophosphate pesticides could weaken acetylcholinesterase, leading to cholinergic toxicity. Carbamate or phosphate pesticides were found to disrupt the equilibrium between acetylcholine synthesis and release, on the one hand, and its hydrolysis, on the other hand, by inhibiting acetylcholinesterase and cause the accumulation of acetylcholine at the synaptic level, resulting in prolonged activation of cholinergic receptors.

Herbicides can result in deformities in plants *via* blocking cell division and photosynthesis or by mimicking natural plant growth hormones. The potential effects of herbicides are strictly dependent on their toxic mode of action and way of application.

## Strategies for new natural product discovery from *Streptomyces*

Although many compounds with biological activity were isolated from *Streptomyces*, only two new classes of antibiotics were introduced in the clinic in the last 30 years. On the other hand, microbial resistance to existing antibiotics is increasing day by day. It is a matter of great concern that humanity may return to the pre-antibiotic era. Antibiotic resistance (AR) is widely considered to be the next global pandemic.

### General consideration for screening new natural products from *Streptomyces*

A large quantity of *Streptomyces* strains having capability to produce bioactive compounds remain unexplored (Genilloud, 2014). Nowadays, new drug discovery has become more difficult, but demands for novel antibiotics in the hospitals have pushed drug screening from nature to be resumed. Some new *Streptomyces* strains have been discovered from new environments. Marine environments have emerged as potential sources during the last decade, as well as soils of deserts, mountain, and icy places. Symbiotic relationships have attracted attentions of the scientific community. Another important perspective is paying attention to natural growth conditions.

After the availability of whole-genome sequencing technique, many cryptic or silent biosynthetic gene clusters (BGCs) in *Streptomyces* were revealed. Among 20–50 biosynthetic pathways for natural products in every *Streptomyces* strain, only a limited number of products have been identified. It still remains a huge challenge to activate these pathways to discover new secondary metabolites from *Streptomyces* (Supplementary Figure S1).

In laboratory/industry, culture conditions for *Streptomyces* growth differ greatly from natural conditions. In natural habitats of *Streptomyces*, natural product production may remain dormant as these substances are related to stress conditions or defense against niche competitors. But most of BGCs are not expressed or slightly expressed under laboratory conditions.

In addition, in laboratory conditions, a majority (>90%) of *Streptomyces* are hard to culture or are even non-culturable. This problem could be solved by using the functional metagenomics approach.

Currently, elicitors are used to manipulate *Streptomyces.* Elicitors can induce differentiation of *Streptomyces* and trigger cryptic antibiotic pathways. Secondary metabolism could also be conditioned by differentiation. The mycelium morphology and different life cycle stages are important for antibiotic production. To improve active cryptic biosynthetic gene clusters and secondary metabolite production, different complementary strategies have been developed, either to improve the screening of new activities or to improve the production of known molecules.

It has been hypothesized that cryptic antibiotic gene clusters that have been inactive may need to be reactivated. Some newer methods were used to make it achievable, such as by adding signaling molecule *N*-acetylglucosamine to the medium for cultivating *S. hygroscopicus, S. collinus, S. clavuligerus, S. griseus*, and *S. venezuelae*, resulting in the activation of antibiotic-producing genes (Rigali et al., 2008). The inclusion of bacterial hormones gamma-butyrolactones (γ-butyrolactones) produced similar results. Alternatively, it was found that adding sub-inhibitory concentrations of lincomycin into the medium caused production of chemicals that are not formed on lincomycin-free medium (Imai et al., 2015).

Aside from the new methods already described, there have been reports of other recent ways used to encourage the identification of new *Streptomyces* compounds. There have also been reports of innovative co-transformation strategies being used in *Streptomyces* sp. (Kallifidas et al., 2012).

Promoter engineering was developed to activate silent BGCs. The Clustered Regularly Interspaced Short Palindromic Repeats-Cas9 (CRISPR-Cas9) gene editing tool was effectively employed to add the kasO promoter (97 base pair) into *S. roseosporus* NRRL 15998. This promoter region then allowed the silent aurR1 gene cluster to be activated, resulting in the synthesis of auroramycin when expressed (Kallifidas et al., 2012).

Despite the fact that whole-genome sequencing was used to identify the gene clusters, heterologous expression of these gene clusters typically goes unnoticed. Scientists employed environmental DNA clones (eDNA), specifically those expressing PKS-type transcription factors, and transplanted them into *S. albus* with the equivalent minimal PKS containing clone. Tetarimycin was eventually produced and isolated in *S. albus* using this approach (Kallifidas et al., 2012).

### Genomic-driven natural product discovery from *Streptomyces*

Recent data suggest that genome mining of large-scale genomic datasets reveals the potential of secretion of various natural products from microbes (Doroghazi et al., 2014; Ju et al., 2015; Pan et al., 2017) with the possibilities of the production of antibiotics too (Bérdy, 2012; Ward and Allenby, 2018). Genome mining is a process that helps perceive the biosynthetic pathway for production of natural compounds (Zhao et al., 2019). This process is dependent on biosynthetic gene clusters (BGCs) for natural products (Medema et al., 2015).

By genome mining approaches *in silico*, it was revealed that *Streptomyces* genomes have 25–70 BGCs, but only a small fraction of these bioactive compounds is synthesized in the laboratory exploiting culture methods (Belknap et al., 2020). Furthermore, genome mining enables the finding of untapped biosynthetic pathways from uncultivated *Streptomyces* hiding in a variety of underexplored habitats, as well as uncharacterized biosynthetic pathways contained in the genomes of cultured *Streptomyces* (Niu, 2018). In this case, knowledge about evolutionary relationships, BGC diversity, and distribution patterns of BGCs are very important. However, it is not easy to analyze the distribution of BGCs in case of *Streptomyces* as these species as prolific producers contain at least 1,000 genomes and 20–70 BGCs on each genome (Ward and Allenby, 2018). A recent study found a cluster of *Streptomyces* BGC by genome mining that have antitumor properties (Belknap et al., 2020). In another study, genome mining is used to identify hidden diversity of clusters of *Streptomyces* having ecological impact (Ward and Allenby, 2018). In *S. coelicolor* genome, cryptic biosynthetic gene clusters were identified by genome mining (Challis, 2014). antiSMASH,^1^ and MIBiG databases^2^ contain reference BGCs. In antiSMASH database, among 39 *Streptomyces* genomes, 1346 BGCs are found. Some BGCs possess conserved regions for production of ectoine, osmotic compatible solute, hopene for prokaryotic membrane stability, siderophores like desferrioxime for iron uptake, melanin and spore pigments, carotenoids like isorenieratene, butyrolactones, geosmin, and methylisoborneol. Chlortetracycline, antimycin, albaflavenone, and ectoine are some of the antibiotics from the BGC clusters by *Streptomyces* (Ward and Allenby, 2018).

Next, with the development of synthetic biology-inspired new strategies, including TAR or Red/ET strategies, large whole BGCs could be directly cloned and expressed successfully in heterologous hosts to produce compounds silent in the native cells (Abbasi et al., 2020), and some *Streptomyces* still act as good candidates as hosts, such as *S. albus* and *S. coelicolor*. *De novo* reconstruction of new BGCs or engineering of pathways is nowadays available and expressed in heterologous chassis cells, allowing more rapid finding new compounds (Alam et al., 2021).

It is unequivocally true that genome mining is a promising area that emphasizes on the biosynthetic pathways of microbes, facilitating the production of antibiotics. Nowadays, there is no alternative to genome mining to quench the thirst of finding new antibiotics.

### Metagenomic approaches for natural product discovery from *Streptomyces*

Metagenomic mining is another effective and practical method for finding natural product biosynthesis pathways. This stage entails collecting uncultured microorganisms from ambient samples, creating a genome library, and then performing function-based or sequence-based screening on the metagenomic library (Alam et al., 2021). However, various hurdles must be overcome before their full biosynthetic potential may be realized. One example is the selection of the vector and host in metagenome mining. BAC vectors, which can hold large DNA inserts harboring complete BGCs, are preferred for metagenomic library construction over cosmid and fosmid vectors. For example, *S. albus* is evaluated for its color phenotypic, whereas sequence-based screening relies on sequence tags. Then, using phylogenetic analysis or PCR-based screening, the clones of interest or BCGs are selected, allowing for faster chemical diversity screening and the discovery of novel antibiotic compounds (Niu, 2018). However, isolating DNA fragments of suitable size from eDNA remains difficult. In addition, *E. coli* and *Streptomyces* spp. are the most typical hosts utilized for library development. Taking into account the metabolic variety of environmental samples, hosts in addition to *E. coli* and actinomycetes need additional investigation.

In total, seven effective epoxyketone proteasome inhibitors and two unusual tryptophan dimer natural products were identified by screening soil samples with conserved sequence tags (Chang et al., 2015; Owen et al., 2015). To enrich the library of scaffold-specific antibacterial products, environmental samples are screened in the presence of a specific antibiotic. This is based on the idea that antibiotic producers must develop a self-defense mechanism. Pekiskomycin, a glycopeptide antibacterial drug with a unique peptide structure, was discovered using PCR and phylogenetic analysis, which help screen potential producers for chemical diversity (Thaker et al., 2013). The *S. albus* metagenomic library led to the discovery of metatricycloene, a novel natural product (Iqbal et al., 2016). It was fascinating that *S. albus* acted as heterologous host to express cadasides (CDE) gene cluster from a soil eDNA sample (Wu et al., 2019).

## Conclusion and future perspectives

Antibiotic resistance is spreading faster than we can develop new antimicrobials and techniques to counteract it. Antibiotic-resistant diseases demand novel drugs. Multidrug resistance is a serious difficulty in treating infectious illnesses, both in the community and in hospitals, and restricts the therapy of bacterial infections. Antibiotic discovery rates decrease drastically after the “golden age” of the 1940s to the 1960s. *Streptomyces* is a promising natural antibiotic manufacturer and bioactive chemical source used to find and make new drugs. Numerous *Streptomyces* species include BGCs that encode chemicals with diverse activity, making them valuable.

However, identifying new antibiotics in *Streptomyces* is difficult because of rediscovering known antibiotics. To avoid rediscovering the same molecules, new multidisciplinary approaches are needed for resuming natural products screening from *Streptomyces*. To improve *Streptomyces* screening, multidisciplinary techniques that integrate methodologies will be critical to unlock hidden pathways and discovering new bioactive chemicals. Biosynthetic pathways from previously unknown *Streptomyces* are now accessible with advances in genomics-driven technologies. It is simple to apply metagenome, pan-genome, and genome mining to these filamentous *Streptomyces* bacteria.

Bioinformatics, heterologous expression, synthetic biology, mass spectrometry (MS), and nuclear magnetic resonance (NMR)-based metabolomics are needed to unravel the intricacy of metabolic pathways in understudied *Streptomyces*. Undoubtedly, advancements in these methods, especially in synthetic biology and artificial intelligence used in bioinformatics fields, will enhance our knowledge of the variety and distribution of these biosynthetic pathways and optimize the biosynthetic potential of chemical weapons against diseases resistant to antimicrobials.

## Author contributions

AL conceived the concept and funds, supervised the work, and validated the results. KA, AM, and SS wrote the original draft of manuscript. KA, JH, and Y-MZ conducted software. SI, CS, and YW conducted validation. YZ contributed to visualization, writing, and data analysis. All authors read and approved the manuscript.

## Footnotes

1
https://antismash-db.secondarymetabolites.org/

2
https://mibig.secondarymetabolites.org/

## Supplementary material

Supplementary Figure 1 General procedures for screening bioactive compounds from *Streptomyces*.
