# Supplementary figures and images for: Streptomyces: The biofactory of secondary metabolites
Source: Front Microbiol. 2022 Sep 29;13:968053. doi: 10.3389/fmicb.2022.968053 (PMC9558229; doi:10.3389/fmicb.2022.968053)

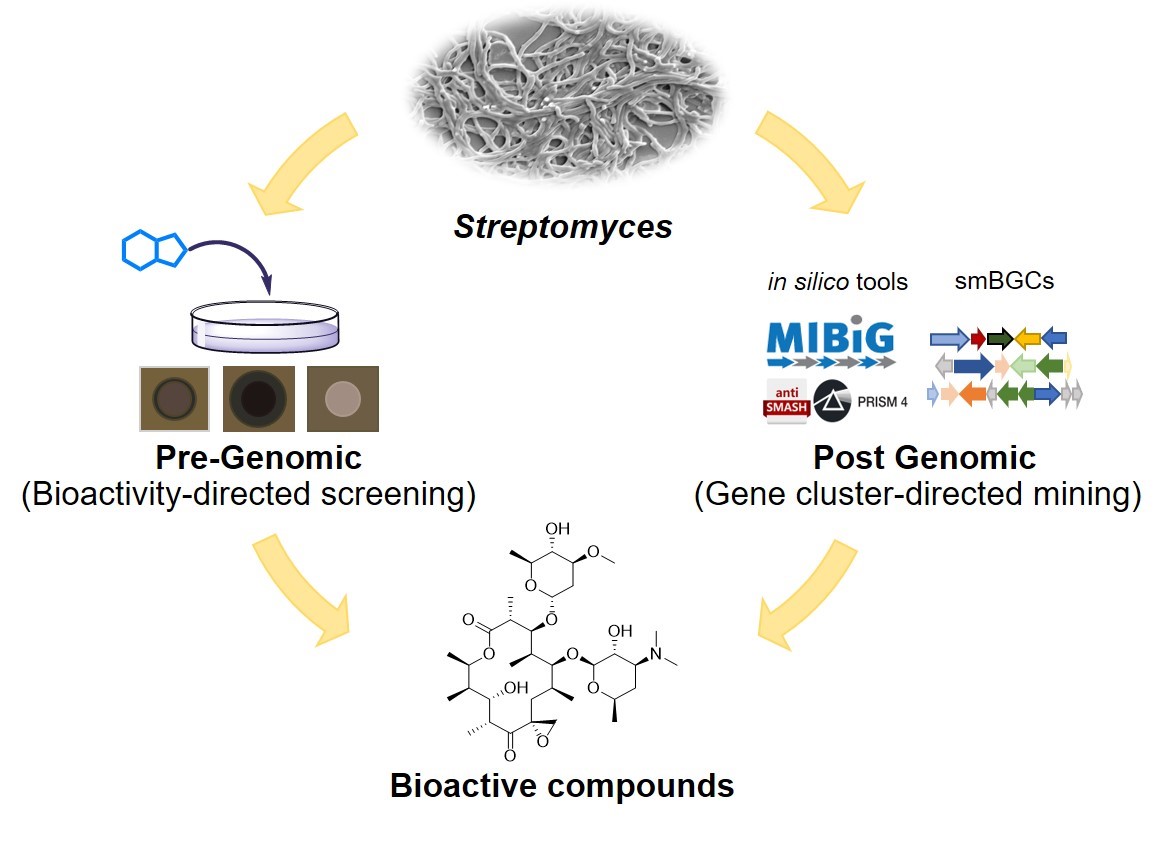

Supplement: Supplementary Figure 1 — General procedures for screening bioactive compounds from Streptomyces. [file Image_1.jpg]
